# Structural Morphology of Molars in Large Mammalian Herbivores: Enamel Content Varies between Tooth Positions

**DOI:** 10.1371/journal.pone.0135716

**Published:** 2015-08-27

**Authors:** Daniela E. Winkler, Thomas M. Kaiser

**Affiliations:** Center for Natural History, University of Hamburg, Hamburg, Germany; Monash University, AUSTRALIA

## Abstract

The distribution of dental tissues in mammalian herbivores can be very different from taxon to taxon. While grazers tend to have more elaborated and complexly folded enamel ridges, browsers have less complex enamel ridges which can even be so far reduced that they are completely lost. The gradient in relative enamel content and complexity of structures has so far not been addressed within a single species. However, several studies have noted tooth position specific wear rates in small mammals (rabbits, guinea pigs) which may be related to individual tooth morphology. We investigate whether differentiated enamel content by tooth position is also to be found in large herbivores. We use CT-scanning techniques to quantify relative enamel content in upper and lower molar teeth of 21 large herbivorous mammal species. By using a broad approach and including both perissodactyls and artiodactyls, we address phylogenetic intraspecific differences in relative enamel content. We find that enamel is highly unevenly distributed among molars (upper M1, M2, M3 and lower m1, m2, m3) in most taxa and that relative enamel content is independent of phylogeny. Overall, relative enamel content increases along the molar tooth row and is significantly higher in lower molars compared to upper molars. We relate this differential enamel content to prolonged mineralisation in the posterior tooth positions and suggest a compensatory function of m3 and M3 for functional losses of anterior teeth.

## Introduction

Most mammalian teeth are composed of three dental tissues: enamel, dentine and cementum. The dentine can be differentiated into primary dentine and secondary dentine. The amount of cementum present is very different from taxon to taxon, but also among tooth positions in the same individual. Some species may lack either cementum or enamel. Such morphologies may be characteristic of a clade (e.g., Xenarthra in whose teeth enamel is completely absent), but are also related to dietary adaptations in various groups and occur in most mammals at least in specific stages of wear.

Specific patterns in distribution of enamel and dentine on the occlusal surfaces of cheek teeth in grazing and browsing species have been noticed in several studies. Archer and Sanson [1], Heywood [2] and Famoso et al. [3] have shown that extant grazers have more and also more elaborated and complexly folded enamel ridges than extant browsers. A similar observation was made by Famoso et al. [3], Kaiser et al. [4], Gailer and Kaiser [5] and Winkler et al. [6], for extinct species, the latter two using 3D-topometry, an approach to quantify proportions of dental tissues on the occlusal surface. These studies have mainly focused on the upper second molar and compared a variety of taxa. So far, dental tissue distribution and enamel complexity has only been quantified on the occlusal surface [3, 7], not analysed over the whole tooth volume. Moreover, it has not been examined whether structural differences in tissue through the entire volume of the tooth exist among teeth of an individual. This question becomes relevant as studies on rabbits [8] and guinea pigs [9] found wear rates specific to tooth position, which might be explained by either differences in structure or in masticatory force on specific tooth positions.

The hardest dental tissue is enamel [10, 11, 12]; hence it should be most resistant to wear. This is universally observed when dentine wears out faster than enamel, forming dentine basins. Under the same dietary regime and loads, teeth with high relative enamel content are therefore expected to wear down slower than teeth with lower relative enamel content.

In the extinct horse *Cormohipparion occidentale*, Kaiser [13] found a decrease in shear-cutting functionality in successive ontogenetic stages of upper P2–M2, which he considered to be compensated for by an increase in function by the M3. This functional compensation was inferred from the orientation of shearing blades and their angle toward the chewing direction. We propose that structural enhancement of the later erupting tooth positions, i.e., higher enamel proportion, would also ensure a longer functionality and compensate for functional loss of anterior tooth positions. In this study, we compare tooth position specific relative enamel content of upper and lower molars (M1, M2, M3/m1, m2, m3) in 21 artiodactyl and perissodactyl taxa. We are testing whether relative enamel content per tooth position is independent from phylogeny and if differences between relative enamel content per tooth position exist. Using only molars ensures a wider comparability between teeth, as premolars are often morphologically different, reduced in size or completely absent. Bovid premolars are for example not comparable to molariform equid premolars. Additionally, we propose that molars have a more homogenous function than premolars, as they are more similar in morphology to each other, and as their positions are closer to the centre of mastication. Along a check tooth row we define the centre of mastication as the area where masticatory contacts are most likely. We use digital tooth models obtained by CT-scanning to quantify tooth crown volume and to differentiate between soft (dentine and cementum) and hard dental tissues (enamel). Only specimens with the upper M3 just erupting or in early wear were selected to ensure that the loss of tooth crown height in the molars is similar in all species. This also ensures coverage of the maximum tooth crown height of M1-M3 within the same individual.

We note that by the time the M3 erupts there is already a substantial loss of tooth crown in the M1 (especially for hypsodont taxa), because it is the first molar to erupt. However, we chose this approach instead of measuring several individuals of different age to exclude inter-individual variations in relative enamel content. Moreover, a study by Winkler & Kaiser [14] has shown that relative enamel content is smaller in the apical crown region of the upper M1 and M2 in *Equus quagga* as compared to the central and basal section of the hypsodont crown. A loss of dental tissue in the apical region would therefore lead to slightly higher measures of enamel proportion over the whole tooth crown. Nevertheless, the difference in enamel proportion between molars was maintained over the whole crown height.

## Material & Methods

All specimens included in this study are curated at the Zoological Museum Hamburg (ZMH) or Museum für Naturkunde Berlin (ZMB, see S1 Table). The sample is comprised of one individual per species. High resolution computed tomography (microCT) scans for all species except Rhinoceroses were obtained at Steinmann-Institut für Geologie, Mineralogie und Paläontologie (Universität Bonn, Germany) on the CT scanner v|tome|× s (GE phoenix|x-ray) with varying kV and μA settings to meet the specific object’s demands. We only chose specimens with complete skulls so that upper and lower teeth belong to occluding dentitions of the same individual. As specimens ranged from approx. 20–40 cm in length, the obtained maximal resolution resulted in voxel sizes between 0.044 mm and 0.237 mm. Due to size limitations of the v|tome|× s, scans of Rhinoceroses (*Diceros bicornis* and *Ceratotherium simum*) were obtained at Universitätsklinikum Hamburg Eppendorf (UKE) on a medical CT with a voxel size of 0.488 mm (see S1 Table for exact voxel size of each specimen).

The software VG StudioMax 2.1 (Volume Graphics, Heidelberg) was used for reconstruction of virtual models and further processing. Based on preservation and completeness of the tooth rows, we analysed either the right or left occluding dentition. Details are given in Table 1. First, each tooth was recreated with all dental tissues (enamel, dentin and cementum) as a voxel model using manual and automatic segmentation tools. All models were cut at the base of the crown by fitting a clipping plane through the cementoenamel junction. Then only the voxels above the clipping plane were selected to create models of the tooth crown. Next, models composed of enamel only were created by segmenting the mineralised tissue with grey thresholding and the region grower tool, which allows to select structures of the same material properties within a defined three-dimensional space. All segmentation results were carefully revised, as enamel densities change through the tooth crown and hence the whole enamel cannot be segmented automatically. If necessary the selected area was corrected manually from slice to slice. Volumes of the enamel model and full tooth model were taken directly from the properties of these models as displayed in VG StudioMax. We then calculated relative enamel content per tooth crown by dividing the enamel volume by total volume of the respective tooth.

**Table 1:** Enamel proportion for each tooth position.

				enamel proportion per tooth					
				upper			lower		
species	order	family	side	M1	M2	M3	m1	m2	m3
*Antilocapra americana*	Artiodactyla	Antilocapridae	left	22.27%	26.49%	30.43%	23.73%	20.97%	33.01%
*Alcelaphus buselaphus*	Artiodactyla	Bovidae	left	29.72%	32.07%	34.27%	32.11%	36.39%	40.29%
*Antilope cervicapra*	Artiodactyla	Bovidae	right	30.07%	38.47%	40.99%	33.32%	39.64%	43.21%
*Antidorcas marsupialis*	Artiodactyla	Bovidae	left	20.50%	23.37%	28.02%	28.11%	31.38%	26.37%*
*Aepyceros melampus*	Artiodactyla	Bovidae	right	34.16%	36.75%	45.61%	31.02%	37.63%	44.98%
*Camelus bactrianus*	Artiodactyla	Camelidae	right	25.96%	30.23%	44.85%	29.93%	28.28%	39.54%
*Capra ibex*	Artiodactyla	Bovidae	right	15.10%	15.43%	19.12%	31.81%	36.24%	42.11%
*Ceratotherium simum*	Perissodactyla	Rhinocerotidae	left	19.44%	21.90%	39.34%	22.19%	29.86%	45.16%
*Connochaetes taurinus*	Artiodactyla	Bovidae	right	20.08%	24.60%	26.86%	21.03%	26.49%	29.64%
*Diceros bicornis*	Perissodactyla	Rhinocerotidae	right	17.50%	18.13%	25.77%	20.80%	20.39%	23.36%
*Damaliscus pygargus*	Artiodactyla	Bovidae	right	28.22%	30.83%	30.52%	31.44%	33.06%	34.39%
*Elaphurus davidianus*	Artiodactyla	Cervidae	right	28.02%	33.54%	33.27%	31.35%	40.83%	49.24%
*Hemitragus jemlahicus*	Artiodactyla	Bovidae	left	28.09%	29.03%	29.14%	28.09%	32.15%	32.13%
*Kobus ellipsiprymnus*	Artiodactyla	Bovidae	right	22.06%	31.22%	28.82%	23.66%	29.99%	32.93%
*Lama glama*	Artiodactyla	Camelidae	left	36.23%	38.60%	48.77%	21.67%	25.19%	32.09%
*Lama huanachus*	Artiodactyla	Camelidae	left	24.22%	30.01%	35.39%	42.82%	38.88%	44.22%
*Litocranius walleri*	Artiodactyla	Bovidae	right	24.20%	27.62%	27.23%	35.31%	38.54%	40.99%
*Naemorhedus goral*	Artiodactyla	Bovidae	left	27.31%	31.68%	30.34%	27.78%	38.22%	39.73%
Ozotoceros *bezoarticus*	Artiodactyla	Cervidae	right	29.13%	35.95%	41.24%	37.54%	39.53%	44.22%
*Redunca fulvorufula*	Artiodactyla	Bovidae	right	35.68%	40.89%	44.24%	49.27%	55.80%	49.45%
*Rangifer tarandus*	Artiodactyla	Cervidae	right	23.71%	32.34%	32.41%	18.37%	24.69%	31.40%

* = The lower m3 of *Antidorcas marsupialis* was not fully mineralised and has hence very low enamel content.

### Statistics

As all data is available as percentages, we first applied an arcsine-transformation in order to transform them to normalise the data [15]. We then performed a phylogenetic MANOVA with species as the independent variable and relative enamel content per tooth position as the dependent variables, using the package geiger version 2.0.3 [16] in R version 2.15.1 [17], which simulates new sets of dependent variables on the phylogenetic tree under a Brownian-motion model. To test for the effects of phylogenetic relatedness we use a tree with branch-lengths (see S1 Fig) including all our tested species which is extracted from the supertree of Bininda-Emonds et al. [18]. We chose this procedure because morphological characteristics cannot be considered to vary independently among evolutionarily related taxa and hence could violate assumptions based on independence of variables [19, 20]. To test for normality and heteroscedasticity of the data, we applied the Shapiro-Wilk normality test [21] and a robust Brown-Forsythe Levene-type [22] test as well as Bartlett’s test [23]. The packages used are ‘stats’ version 3.3.0 [17] and ‘car’ version 2.0.25 [24]. Results show (see S2 Table and S3 Table for test statistics) that our data are normally distributed and homoscedastic. Subsequently, we performed a one-way ANOVA [25, 26]coupled with a post-hoc Tukey test [27, 28, 29] to test for an underlying pattern in tooth position specific relative enamel content. All data was pooled for this approach. We further tested for significant differences between enamel proportion in upper and lower dentitions.

## Results

**Fig 1:**
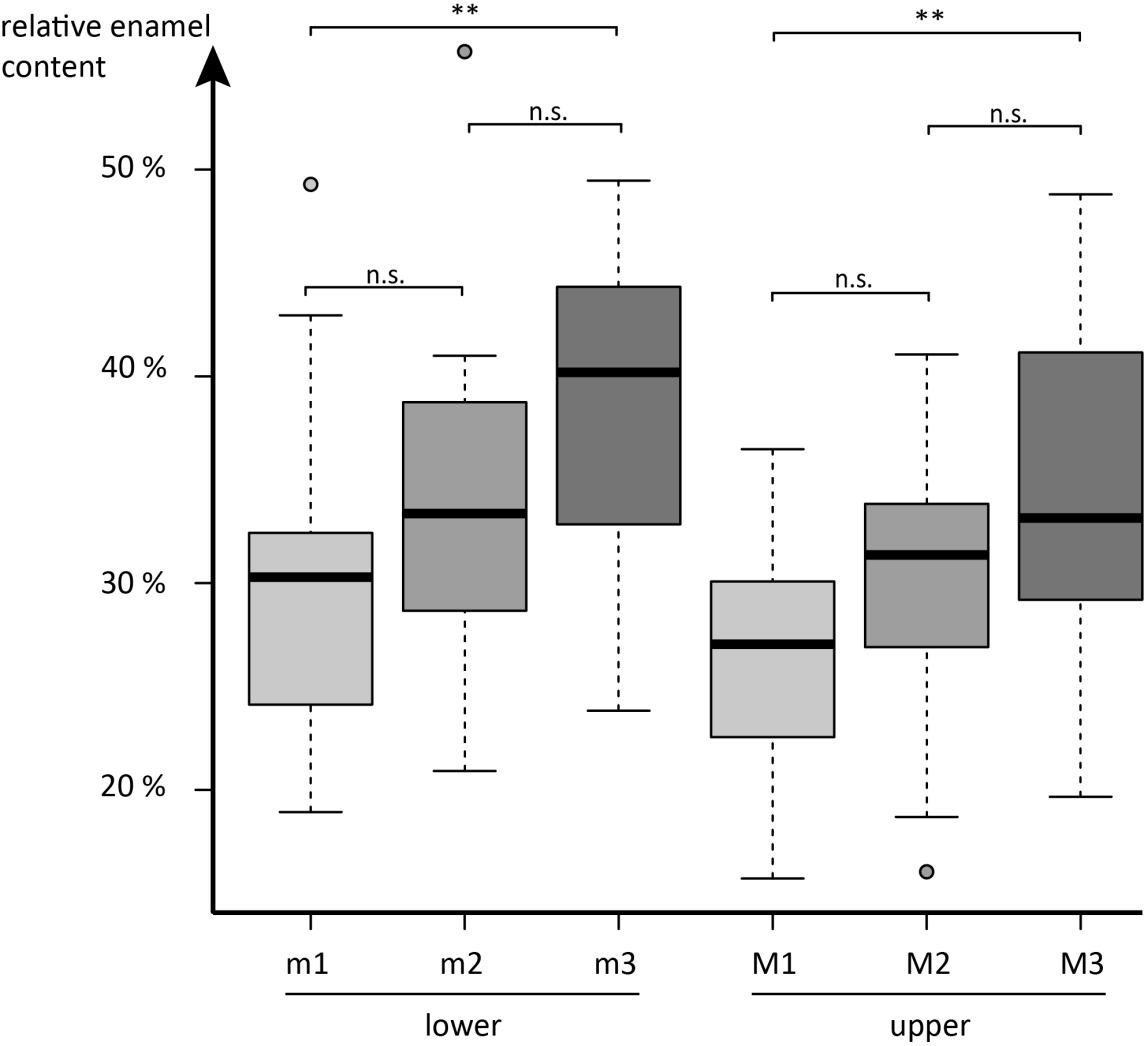
Boxplots of overall relative enamel content per tooth position. *: *p* = 0.05, ***: *p* = 0.001, n.s. = not significant (all p-values from post-hoc Tukey test).

**Fig 2:**
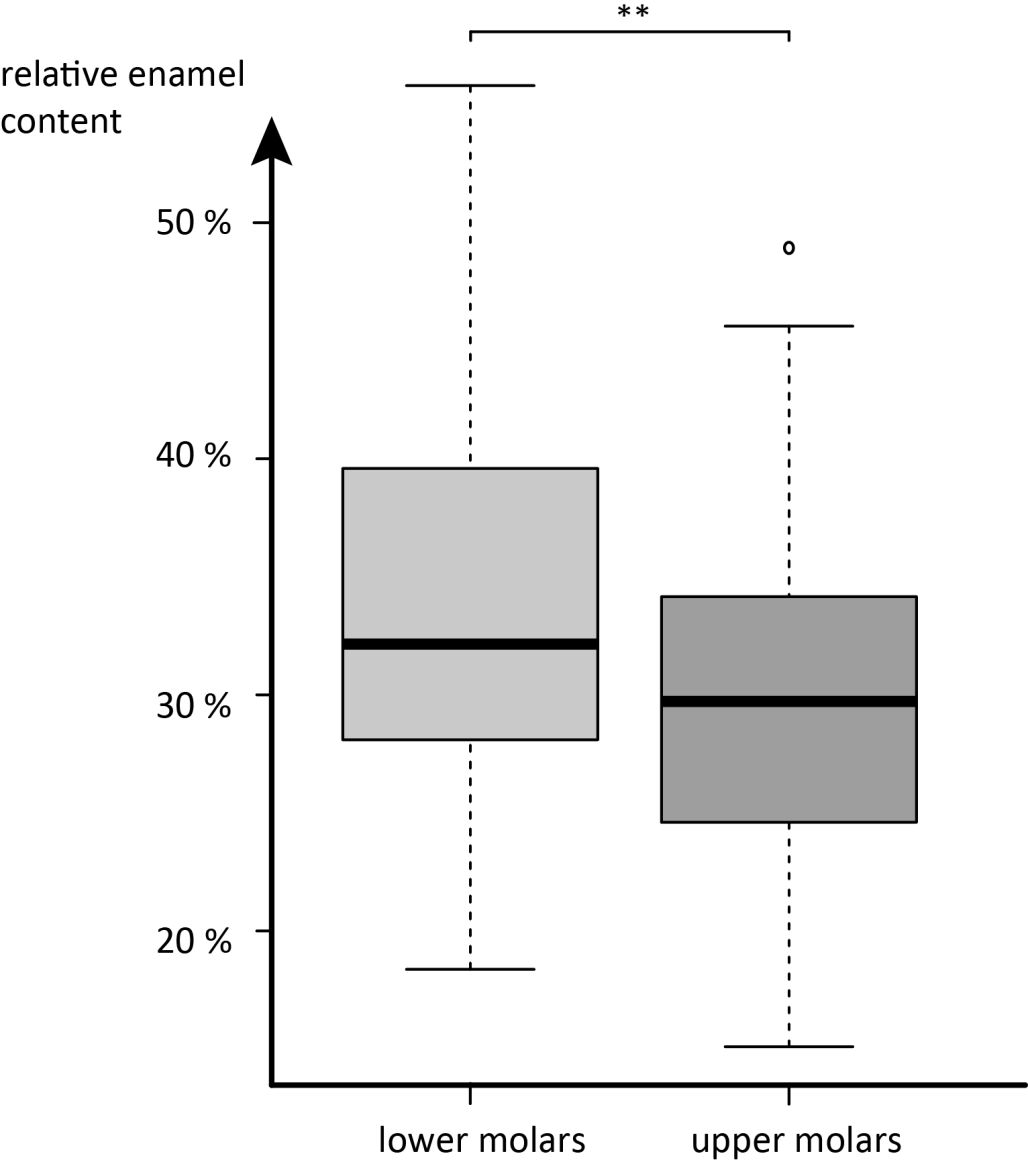
Boxplots of pooled relative enamel content of all upper versus all lower molars. *: p = 0.05, ***: p = 0.001, n.s. = not significant (all p-values from post-hoc Tukey test).

**Fig 3:**
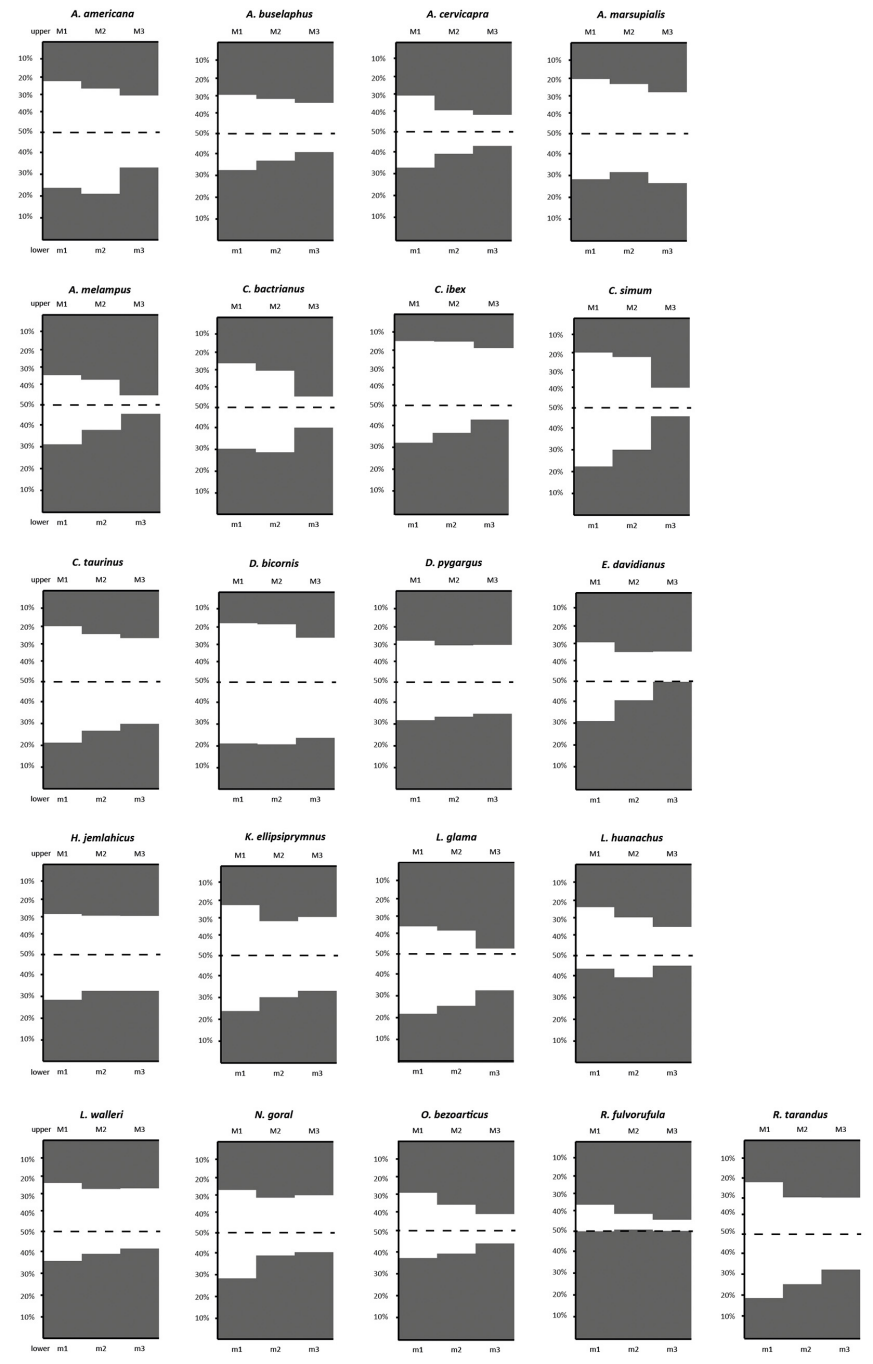
Histograms of relative enamel content for upper and lower molars of each species.

We find no significant influence of phylogenetic affiliation with tooth position specific relative enamel content (Table 2). Between tooth positions, however, significant differences in relative enamel content are detected in an ANOVA and post-hoc Tukey test (Tables 3 and 4). In all individuals investigated, upper M2s had higher relative enamel content than upper M1s, though this observation is not statistically significant. For lower molars, this relation holds true in 17 out of 21 species. Amongst the four deviating species, only *Antilocapra americana* showed a substantially smaller enamel proportion in lower m2 compared to lower m1 (12%), while *Lama huanachus* had 4% less relative enamel content in the lower m2 and the other two had nearly equal relative enamel contents in lower m1 and m2 (compare Table 1: *Camelus bactrianus*, *Diceros bicornis*). In 16 out of 21 cases upper M3s were composed of relatively more enamel than upper M2s, however, in two of them the difference is less than 1%. Upper M3s have significantly higher enamel proportions than upper M1s (*p* = 0.003), but upper M3s do not have significantly higher enamel proportions (*p* = 0.429) than upper M2s (Fig 1, Table 3). None of the five species with smaller relative enamel content in upper M3 than in upper M2 showed substantial differences; three of them even having only 1% less relative enamel content (Table 1). The largest difference was found in *Kobus ellipsiprymnus* with 3% lower enamel proportion in upper M3 than in upper M2. In lower molars, 19 out of 21 species showed larger relative enamel content in lower m3 compared to lower m2 and one had nearly equal relative enamel content (*Hemitragus jemlahicus*). Overall, the higher relative enamel content in lower m3s compared to lower m1s is significant (*p* = 0.002), but not between lower m2s and m3s (*p* = 0.244). *Antidorcas marsupialis* had 16% less relative enamel content per lower m3 volume compared to lower m2. However, the lower m3 was, in contrast to the upper M3, not mineralised at the crown base. In this species the true enamel proportion of the lower m3 could therefore not be determined and it was excluded from the statistical analysis. In general, lower molars have a significantly higher relative enamel content (*p* = 0.008) than their upper antagonists (Fig 2, Tables 3 and 4). See Fig 3 for histograms showing enamel proportion per tooth position for all species investigated.

**Table 2:** Test statistics for the phylogenetic MANOVA. Species was used as the independent variable and relative enamel content per tooth position as the dependent variables. No significant influence of phylogeny was detected. *df* = degree of freedom, *F* = test value, *p* = significance level.

	*df*	Wilks’ lambda	approx. *F*	numerator d*f*	denominator *df*	*p*(>F)	*p*(phy)
group	1	0.80293	0.57267	6	14	0.74592	0.3437

**Table 3:** Results of one-way ANOVA with tooth position or jaw as factor and relative enamel content as the numeric variable. Values in bold indicate a significant interaction (*p* ≤ 0.05). Statistics abbreviations: *df* = degree of freedom, *SumSq* = sum of squares, *MeanSq* = mean of squares, *F* = test value, *p* = significance level.

	ANOVA				
	*df*	*SumSq*	*MeanSq*	*F*	*p*
tooth position	5	0.2384	0.04768	7.789	**0.000**
residuals	119	0.7284	0.00612		
jaw	1	0.0547	0.05474	7.383	**0.008**
residuals	123	0.9121	0.00742		

**Table 4:** Results of Post-hoc Tukey test for multiple comparisons of means with 95% family-wise confidence level. Values in bold indicate a significant difference (*p* ≤ 0.05) for relative enamel content between tooth positions. Statistics abbreviations: *diff* = difference between group means, *lower* = lower end point of the interval, *upper* = upper end point of the interval, *p* = significance level.

		Post-hoc Tukey Test			
Group 1	Group 2	*diff*	*lower*	*upper*	*p*
lower m1	lower m2	0.043	-0.027	0.113	0.486
lower m1	lower m3	0.097	0.026	0.168	**0.002**
lower m2	lower m3	0.054	-0.017	0.125	0.244
upper M1	upper M2	0.046	-0.024	0.116	0.397
upper M1	upper M3	0.091	0.021	0.161	**0.003**
upper M2	upper M3	0.045	-0.025	0.115	0.429
all upper	all lower	-0.042	-0.072	-0.011	**0.008**

## Discussion

We find enamel distribution along the molar tooth row to show a specific pattern, which is independent from phylogeny. Hence we consider the distribution of relative enamel content to be related either to adaptive or developmental processes in the tested taxa. Lower molars show significantly higher relative enamel content than upper molars. Along the molar tooth row, relative enamel content increases in upper and lower jaw from M1 over M2 to M3, though only the difference between M1 and M3 is significant in both upper and lower molars. However, a general tendency for increasing relative enamel content is observed and we propose two hypotheses to explain this observation.

### Functional compensation hypothesis

With advancing age it has been observed that the anterior teeth, especially the premolars and the first molar, wear down to a stage where shear-cutting functionality decreases successively until there are no longer any inner enamel structures exposed on the occlusal surface [30]. Dentine then forms a large basin that may function as a compression basin, but does not provide grinding functionality. The posterior molars M2 and particularly M3 maintain their morphology of distinct enamel ridges much longer. This is obviously related to their later eruption time, as the molars erupt from front to back and hence the M1 has been in wear long before M2 and M3 erupt. However, our results show that in most species analysed, M2 and M3 have higher relative enamel content than the M1. This relation is found both for upper and lower dentitions. Teeth built from less dentine and more enamel are thus expected to be more robust to wear and maintain function until a later life stage. We interpret this maintained functionality as a compensation for functional loss in shear cutting and grinding in anterior teeth and conclude that the animals shift their masticatory centre towards the rear of the dentition.

### Molar retention hypothesis

Smith [31] stated that molars emerge from front to back; and to our knowledge no mammal taxon exists (where identification of tooth positions is possible) which would challenge this rule. In the foetus, molar tooth development starts from the front to the back, with the anterior teeth controlling the size of their posterior neighbour [32]. The onset of their development is, however, thought to be nearly simultaneous. The possible time for molar development is hence associated with tooth eruption, and higher enamel content in upper and lower M2s and M3s could therefore be related to a prolonged amelogenesis. The onset of M3 eruption is very different between taxa. While the first two molars erupt relatively fast, it may take between 8 and 12 month for pronghorns [33], 16 month in cervids [34], 24 months in goats and sheep [35, 36] and 26–33 month in wildebeest [37] for the third upper and lower molar to erupt. In larger species, like *Ceratotherium simum*, the eruption of M3 does not take place before the 4^th^ year [38]. In consequence it would be plausible that longer retention would relate to longer activity of enamel secretion and thus increased likelihood that a higher enamel content develops in posterior cheek teeth.

## Conclusion

Enamel mineralisation is usually finished when the teeth start to wear. Final maturation of the mineralised enamel however does continue after the completion of tooth eruption. For human third molars, Driessens et al. [39] suggest a post-eruptive maturation of the outer enamel up to a depth of 10–30μm. In domestic horses, Hoppe et al. [40] found that the final mineralisation of enamel continued 6–12 month after the beginning of tooth eruption. As the approach for this study required examining relatively young animals with the upper M3 just erupting or in early wear (to prevent tooth material loss in anterior molars), we can expect enamel mineralisation in M3s not to be complete at the time of death. Less mineralised enamel has a lower density than fully mineralised enamel. In the CT image, less mineralised enamel has a similar grey scale as dentine or cementum and will therefore be hard to distinguish from the softer dental tissues. We thus expect to have underestimated the true enamel content in a mature M3s, which in fact would be even higher than measured in the pre-mature teeth selected, in order to keep this study free of the effects of intraspecific variability. The gradients we find here are independent of phylogeny and likely to be even more strongly expressed during an animal’s lifespan than can be shown here. However, as we only analysed one specimen per species, we cannot draw conclusion on subtle differences that might exist between taxa.

We further note that in most taxa analysed, the lower molars show a significantly higher enamel content than the upper molars. It seems plausible that this observation is related to the phenomenon of positive anisodonty (upper teeth wider than lower teeth) in most mammals that could be derived from basic relationships imposed by position and geometry. This phenomenon has been discussed for a long time; however, no conclusive evolutionary model has been forwarded to relate morphology with functional constraints. Kaiser and Fortelius [41] proposed the hypothesis that an arrangement maximizing sharpness in upper teeth would be superior to one maximizing sharpness in lowers, which are more influenced by food abrasion between occlusal events, because of the action of gravity. Kaiser and Fortelius [41] further conclude, that “once the polarity is set, much of the rest will probably follow from basic geometry, as the inverted pestle-and-mortar system of positive anisodonty will of geometric necessity create deeper cutting blades on the mortar part, i.e., the upper tooth, and these blades will be kept sharp by attrition against the lowers.”

Higher enamel proportions in lower molars mean lower proportions of dentine in turn. In functional aspects this might either translate to smaller dentine basins, or wider enamel bands on the occlusal surface. In order to add to our understanding of the phenomenon of anisodonty in mammals and its underlying functionality, it would be desirable to test absolute volumes of dental tissues. Combined with our data, this would allow for testing of the “inverted mortar and pestle” hypothesis by Kaiser and Fortelius [41], that lower molars would have functionally evolved into maximum wear resistance, while upper molars would rather be adapted to the specific needs of efficient shear cutting action.

## Supporting Information

S1 Fig Phylogenetic tree adapted from Bininda-Emonds et al. [18]. (TIF)

S1 Table Voxel size per specimen. The scans included the whole upper or lower jaw. If possible, mandibles were separately scanned. As size is very different between specimens, kV and μA settings vary. Hence the obtained resolution is not always the same.
(DOCX)

S2 Table Results of Shapiro-Wilk normality test testing against assumption of normality. Each variable (enamel content per tooth position) rejects the hypothesis; hence none violates the assumption of normality. *W* = test value, *p* = significance level.
(DOCX)

S3 Table Results of robust Brown-Forsythe Levene-type test Bartlett’s test. There is no significant difference in variances, hence we assume variance homogeneity. *t*, *K-squared* = test value, *p* = significance level.
(DOCX)
