# Supplementary figures and images for: Structural Morphology of Molars in Large Mammalian Herbivores: Enamel Content Varies between Tooth Positions
Source: PLoS One. 2015 Aug 27;10(8):e0135716. doi: 10.1371/journal.pone.0135716 (PMC4551798; doi:10.1371/journal.pone.0135716)

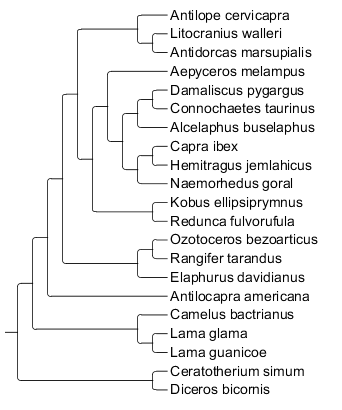

Supplement: S1 Fig — (TIF) [file pone.0135716.s001.tif]
