# Supplementary material for: Structural Morphology of Molars in Large Mammalian Herbivores: Enamel Content Varies between Tooth Positions
Source: PLoS One. 2015 Aug 27;10(8):e0135716. doi: 10.1371/journal.pone.0135716 (PMC4551798; doi:10.1371/journal.pone.0135716)
Supplement: S1 Table — The scans included the whole upper or lower jaw. If possible, mandibles were separately scanned. As size is very different between specimens, kV and μA settings vary. Hence the obtained resolution is not always the same. (DOCX) [file pone.0135716.s002.docx]

|  |  | Voxel size [mm] | |
| --- | --- | --- | --- |
| species | ID | upper jaw | lower jaw |
| *Aepyceros melampus* | ZMH-10162 | 0.09399600 | 0.08943600 |
| *Alcelaphus buselaphus* | ZMH-7487 | 0.15266193 | 0.14250546 |
| *Antidorcas marsupialis* | ZMH-9349 | 0.08393121 | 0.09714112 |
| *Antilocapra americana* | ZMH-8268 | 0.11173152 | 0.10878870 |
| *Antilope cervicapra* | ZMH-5809 | 0.09847700 | 0.08734900 |
| *Camelus bactrianus* | ZMH-1870 | 0.2379870 | 0.23182495 |
| *Capra ibex* | ZMH-6983 | 0.10357185 | 0.09550173 |
| *Ceratotherium simum* | ZMH-2552 | 0.4882812 | 0.4882812 |
| *Connochaetes taurinus* | ZMH-6777 | 0.18507203 | 0.14641845 |
| *Damaliscus pygargus* | ZMB_Mam_55243 | 0.12308109 | 0.13124571 |
| *Diceros bicornis* | ZMH-9379 | 0.4882812 | 0.4882812 |
| *Elaphurus davidianus* | ZMB_Mam_75425 | 0.16599028 | 0.15885973 |
| *Hemitragus jemlahicus* | ZMH-7747 | 0.10880216 | 0.09990503 |
| *Kobus ellipsiprymnus defassa* | ZMH-447 | 0.16947030 | 0.15275939 |
| *Lama glama* | ZMH-8254 | 0.11817878 | 0.10593192 |
| *Lama huanachus* | ZMH-286 | 0.12180461 | 0.10851343 |
| *Litocranius walleri* | ZMB_Mam_39663 | 0.09603801 | 0.06442925 |
| *Naemorhedus goral* | ZMH-478 | 0.07317639 | 0.07750785 |
| *Redunca fulvorufula* | ZMH-7800 | 0.09426996 | 0.09426996 |
| *Ozotoceros bezoarticus* | ZMH-7632 | 0.08039447 | 0.07199855 |
| *Rangifer tarandus* | ZMH-7104 | 0.12239885 | 0.12049037 |

**S1 Table. Voxel size for each specimen.** The scans included the whole upper or lower jaw. If possible, mandibles were separately scanned. As size is very different between specimens, kV and µA settings vary. Hence the obtained resolution is not always the same.
