# Supplementary material for: Structural Morphology of Molars in Large Mammalian Herbivores: Enamel Content Varies between Tooth Positions
Source: PLoS One. 2015 Aug 27;10(8):e0135716. doi: 10.1371/journal.pone.0135716 (PMC4551798; doi:10.1371/journal.pone.0135716)
Supplement: S2 Table — Each variable (enamel content per tooth position) rejects the hypothesis; hence none violates the assumption of normality. W = test value, p = significance level. (DOCX) [file pone.0135716.s003.docx]

|  | Relative enamel content of | | | | | |
| --- | --- | --- | --- | --- | --- | --- |
|  | lower m1 | lower m2 | lower m3 | upper M1 | upper M2 | upper M3 |
| *W* | 0.952 | 0.945 | 0.951 | 0.979 | 0.959 | 0.958 |
| *p* | 0.369 | 0.279 | 0.357 | 0.911 | 0.503 | 0.473 |

**S2 Table. Results of Shapiro-Wilk normality test testing against assumption of normality.** Each variable (enamel content per tooth position) rejects the hypothesis; hence none violates the assumption of normality. *W* = test value, *p* = significance level.
