# Supplementary material for: Structural Morphology of Molars in Large Mammalian Herbivores: Enamel Content Varies between Tooth Positions
Source: PLoS One. 2015 Aug 27;10(8):e0135716. doi: 10.1371/journal.pone.0135716 (PMC4551798; doi:10.1371/journal.pone.0135716)
Supplement: S3 Table — There is no significant difference in variances, hence we assume variance homogeneity. t, K-squared = test value, p = significance level. (DOCX) [file pone.0135716.s004.docx]

| Brown-Forsythe test | | Bartlett's test | |
| --- | --- | --- | --- |
| *t* | *p* | *K-squared* | *p* |
| 0.3047 | 0.909 | 0.535 | 0.970 |

**S3 Table. Results of robust Brown-Forsythe Levene-type test Bartlett’s test.** There is no significant difference in variances, hence we assume variance homogeneity. *t*, *K-squared* = test value, *p* = significance level.
